# Structural and Computational Insights into a Blebbistatin-Bound Myosin•ADP Complex with Characteristics of an ADP-Release Conformation along the Two-Step Myosin Power Stoke

**DOI:** 10.3390/ijms21197417

**Published:** 2020-10-08

**Authors:** Wiebke Ewert, Peter Franz, Georgios Tsiavaliaris, Matthias Preller

**Affiliations:** 1 Institute for Biophysical Chemistry, Structural Bioinformatics and Chemical Biology, Hannover Medical School, 30625 Hannover, Germany; ewert.wiebke@mh-hannover.de; 2 Institute for Biophysical Chemistry, Cellular Biophysics, Hannover Medical School, 30625 Hannover, Germany; franz.peter@mh-hannover.de (P.F.); tsiavaliaris.georgios@mh-hannover.de (G.T.); 3 Department of Natural Sciences, University of Applied Sciences Bonn-Rhein-Sieg, 53359 Rheinbach, Germany

**Keywords:** myosin, molecular motor, ADP release, blebbistatin, structural biology, cytoskeleton, molecular dynamics simulations, force generation

## Abstract

The motor protein myosin drives a wide range of cellular and muscular functions by generating directed movement and force, fueled through adenosine triphosphate (ATP) hydrolysis. Release of the hydrolysis product adenosine diphosphate (ADP) is a fundamental and regulatory process during force production. However, details about the molecular mechanism accompanying ADP release are scarce due to the lack of representative structures. Here we solved a novel blebbistatin-bound myosin conformation with critical structural elements in positions between the myosin pre-power stroke and rigor states. ADP in this structure is repositioned towards the surface by the phosphate-sensing P-loop, and stabilized in a partially unbound conformation via a salt-bridge between Arg131 and Glu187. A 5 Å rotation separates the mechanical converter in this conformation from the rigor position. The crystallized myosin structure thus resembles a conformation towards the end of the two-step power stroke, associated with ADP release. Computationally reconstructing ADP release from myosin by means of molecular dynamics simulations further supported the existence of an equivalent conformation along the power stroke that shows the same major characteristics in the myosin motor domain as the resolved blebbistatin-bound myosin-II·ADP crystal structure, and identified a communication hub centered on Arg232 that mediates chemomechanical energy transduction.

## 1. Introduction

**Figure 1:**
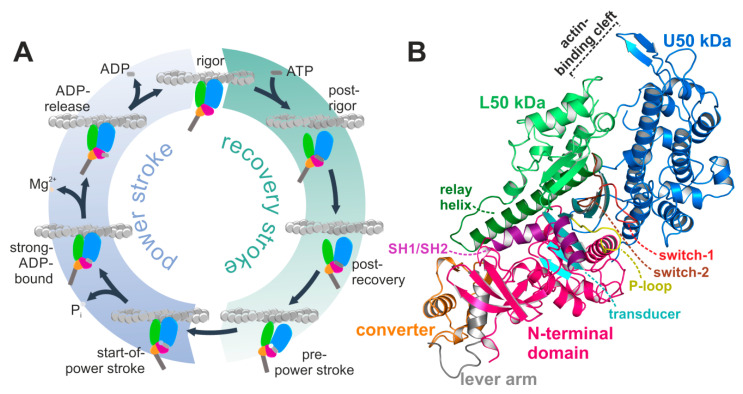
The actomyosin system. (**A**) Scheme of the actomyosin motor cycle. The myosin motor is subject to conformational changes of the subdomains upon interaction with both the actin filament and the nucleotide adenosine triphosphate (ATP), as well as during nucleotide hydrolysis, and release of its hydrolysis products inorganic phosphate (P_i_) and adenosine diphosphate (ADP) to eventually produce force in a two-step mechanism. Color code: subdomains N-terminal domain (pink), U50 kDa (blue), L50 kDa (green), converter (orange), lever arm (grey). (**B**) Structure of the nucleotide-free *Dd* myosin-II motor domain, possessing a closed actin-binding cleft, a twisted transducer (cyan), and the converter (orange)/lever arm (grey) in the down position. The active site with the nucleotide sensors P-loop (yellow), switch-1 (red), and switch-2 (brown) is empty and prepared for ATP binding.

Myosin is a ubiquitously expressed adenosine triphosphate (ATP)-driven motor protein, producing force and directed motion along cytoskeletal actin filament tracks by converting chemical energy from ATP hydrolysis into mechanical work. A multitude of cellular processes is driven by these molecular machines, ranging from cell motility and division to cargo transport, endocytosis, and contraction of cardiac, skeletal, and smooth muscles. Despite their specialized cellular functions, all members of the myosin superfamily share a common multistep mechanism of force generation—the chemomechanical actomyosin motor cycle (Figure 1A) [1,2,3]. The states of that cycle are defined by the status of distinct structural elements, which are allosterically interconnected and remotely affect each other. Pivotal to this allosteric communication is the central seven-stranded β-sheet (the transducer) (twisted in the rigor state, but untwisted in the pre-power stroke state) in the myosin motor domain, which couples the large actin-binding cleft (closed while strongly attached to F-actin, and open in actin-detached states) with the active site phosphate-sensing elements—the P-loop, switch-1 and switch-2—(closed in the hydrolysis-competent pre-power stroke state, but partially or fully opened in all other states) as well as through the relay helix (bent in the pre-power stroke state, while straight in the rigor state) and SH1/SH2-region with the mechanical converter and lever arm (down position in the rigor state, and up position in the pre-power stroke state). We and others have shown that mutations in these key regions disturb the motor function by interfering with chemomechanical coupling pathways [4,5,6,7,8,9].

The myosin motor is thus subject to substantial conformational changes along that cycle, traversing through different actin-attached and –detached states, eventually amplifying them to a large scale rotation of the converter domain and swinging of the adjacent lever arm in the force-generating power stroke [10]. Many of the subprocesses have been extensively studied in the past, and newly observed conformations, obtained through cryo-electron microscopy (cryoEM) [11,12,13] or crystallization with native and artificial ligands [14,15,16,17] that trapped myosin in a specific state, have been assigned as states of the actomyosin cycle according to the status of the characteristic structural elements in the myosin motor domain, thus building the basis for the motor function. Numerous studies have significantly contributed to our understanding of the mechanism of the recovery stroke [18,19,20,21,22,23], which primes the myosin motor for force production while dissociated from the actin filament. However, critical details about the molecular events of the actomyosin motor cycle, which directly contribute to force production and product release, i.e., the myosin power stroke, are still incomplete.

The power stroke is initiated by ATP hydrolysis, followed by reattachment of the myosin motor in the pre-power stroke state to its actin filament track, coupled to conformational changes that finally lead to the release of the hydrolysis products and to force generation. The exact sequence of events is critically discussed in the field, and several models have been proposed for the conformational states involved in the power stroke [24,25,26,27]. The conformational changes required for force production are assumed to be driven by actin reattachment and the sequential release of inorganic phosphate (P_i_), Mg^2+^, and adenosine diphosphate (ADP). Crystal structures in the pre-power stroke state and the nucleotide-free rigor-like state revealed that the central transducer undergoes a twisting motion of the β-strands upon rebinding of myosin to the actin filament, linked to closure of the large cleft between subdomains U50 and L50 kDa to allow strong actin binding [28] (Figure 1B). Kinetic analysis predicts phosphate release from the myosin motor domain directly after ATP hydrolysis and prior to closure of the large actin-binding cleft, which initiates the weak-to-strong actin binding [25]. Several mechanisms for P_i_ release have, however, been postulated, both prior or subsequent to cleft closure, suggesting that P_i_-release occurs either through a side door [10,29], formed by repositioning of switch-1, or a backdoor by switch-2 opening [25,30]. Comparison of the recent Mg^2+^·ADP-bound actin-attached cryoEM models with the nucleotide-free actomyosin rigor complex suggests a two-step mechanism of the power stroke [11,12,13,31]. According to these studies, the larger first step of the power stroke is linked to actin-induced cleft closure and P_i_ release, while a second, smaller stroke with an approximately 10° rotation of the converter might occur during the release of Mg^2+^·ADP [31]. However there are discrepancies about the degree of converter rotation in the two steps of the power stroke and due to the resolution limitations of the cryoEM models, details regarding the molecular mechanisms for ADP release remain speculative.

We therefore present here high-resolution structural insights into a novel conformation of the myosin-II motor domain that was obtained using the well-characterized small molecule myosin-II inhibitor blebbistatin [32]. This myosin-II crystal structure reveals characteristic structural features of the chemomechanical transducing elements—i.e., the central transducer, actin-binding cleft, active site switches, relay helix, SH1/SH2-region, converter and lever arm—in conformations between the myosin pre-power stroke and rigor positions, with the nucleotide partially unbound. These observations suggest that the obtained crystal structure in the presence of blebbistatin might resemble a myosin conformation involved in ADP release towards the end of the power stroke. ADP release is key for the regulation of the mechanical motor function of myosin as it controls the fraction of time the motor stays attached to the actin filament. A series of complementing molecular dynamics simulation techniques further provided a mechanistic framework for nucleotide release from the myosin motor domain, and corroborated that an equivalent myosin conformation plays a role during ADP release and the second step of the power stroke.

Blebbistatin was previously shown to inhibit myosin-II motor function by blocking the motor domain in the pre-power stroke state [14,33,34]. Our results, however, are in line with other studies using X-ray diffraction of muscle thick filaments [35,36] and negative stain electron microscopy together with kinetic analysis [37], which reported blebbistatin to stabilize an additional myosin state in the presence of ADP. These studies postulated that blebbistatin traps myosin in a state at the start of the power stroke in the presence of ADP. In contrast, our high-resolution crystal structure reveals a myosin-II·ADP·blebbistatin structure that shows high similarities with a putative ADP releasing conformation of the motor cycle during the second step of the power stroke near the rigor conformation, underpinning that the inhibitory mechanism of blebbistatin is much more complex than earlier assumed [38].

## 2. Results

### 2.1. Structural Features of the Blebbistatin-Bound Myosin-II ADP Conformation

**Figure 2:**
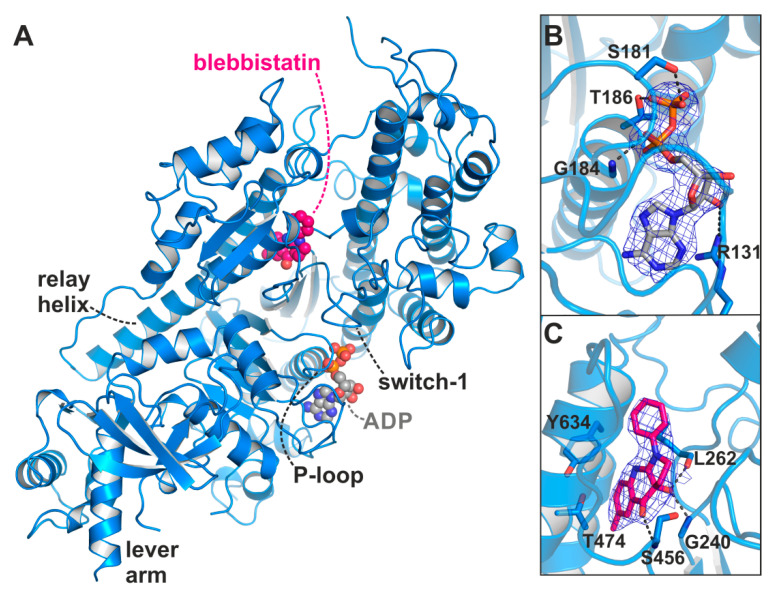
X-ray crystal structure of the *Dd* myosin-II motor domain in complex with ADP and blebbistatin. (**A**) Overview of the crystal structure in the blebbistatin-bound ADP-release conformation. The P-loop together with the bound ADP (grey) is shifted towards the surface. Blebbistatin (magenta) is buried in the known allosteric binding pocket at the apex of the large actin-binding cleft. (**B**) The 2F_o_-F_c_ density map of ADP in its new binding position. The map was contoured at 1.0 σ. Note that the β-phosphate has rotated away from the P-loop. (**C**) 2F_o_-F_c_ density map of blebbistatin at the apex of the actin-binding cleft and near the relay helix. The map was contoured at 1.0 σ.

In a series of crystallization trials using the myosin-II inhibitor blebbistatin, we obtained a previously unknown conformation of myosin in the presence of ADP. We solved the crystal structure of the *Dd* myosin-II motor domain in complex with blebbistatin and ADP to a resolution of 2.58 Å (Figure 2A, see Table 1 for data statistics). The crystals grew in the P2_1_2_1_2_1_ space group and showed unambiguous electron density for the protein residues, the nucleotide, and blebbistatin (Figure 2B,C). This novel myosin conformation differs markedly from the current crystallographically resolved states of the actomyosin cycle. The hallmarks of the unique structure are a converter position close to the rigor down position, rearrangements of the active site switches closer together, and a large shift of the P-loop together with the bound ADP towards the protein surface, while the β-phosphate of ADP lost most of its coordination with protein residues. The positions of the active site switches (closed with the P-loop shifted towards the surface), and the converter/lever arm (near down position), as well as the status of the actin-binding cleft (almost closed), the relay helix (straight), and the transducer (almost fully twisted) suggest that the structure resembles a myosin conformation towards the end of the power stroke that promotes ADP release. Blebbistatin was found in the same binding site as observed in the crystal structure of the myosin-II pre-power stroke state [14], and interacts with residues of the relay and W-helices, as well as active site switches in close proximity to β-strands 5 to 7 of the transducer (Figure 2C).

#### 2.1.1. Comparison with the Myosin-II Rigor and Pre-power Stroke States

As a reference, we additionally crystallized the nucleotide-free apo *Dd* myosin-II motor domain, and solved the structure to a resolution of 1.88 Å (Figure 1B, see Table 1 for data statistics). The overall conformation highly resembled the previously solved structure of a myosin-II-dynamin fusion construct [39] (root mean square deviation (rmsd) 0.54 (chain A) and 0.57 (chain B)), which was assigned earlier as the *Dd* myosin-II rigor-like state, with the converter in the down position and the relay helix straightened. A twisted transducer in our crystal structure primed the nucleotide-free active site for binding of ATP, with an intact critical salt-bridge between switch-1 (residue Arg238) and switch-2 (residue Glu459), which has been suggested to play a central role during P_i_ release [25]. The P-loop (^179^GESGAKT^186^) moved away from the switches together with the N-terminal domain. As observed with the dynamin-fusion rigor-like myosin-II structure (pdb: 2aka), the large actin-binding cleft of our rigor-like myosin-II crystal structure was closed both in the inner (10.7 Å between the C_α_ atoms of residues Ser272 and Ser465) and outer cleft (10.2 Å between the C_α_ atoms of residues Ser416 and Lys589) as compared to the *Dd* myosin-II pre-power stroke state structure (inner cleft: 12.6 Å, outer cleft: 13.4 Å), but to a smaller extent than rigor-like structures of other myosin classes such as myosin-V (inner cleft: 9.6 Å, outer cleft: 7.2 Å) [40]. Indeed, this is a well-documented feature of *Dd* myosin-II [40].

**Figure 3:**
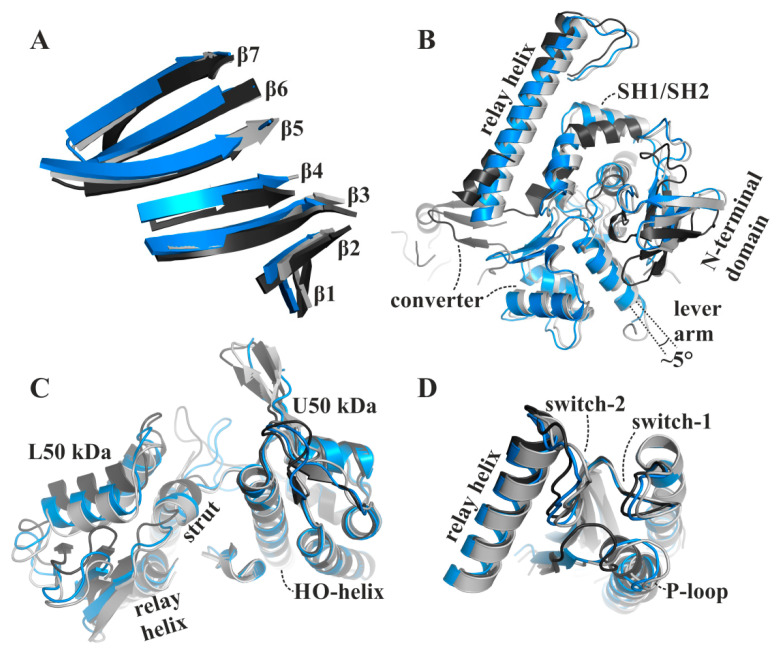
The blebbistatin-bound myosin-II·ADP structure (blue) feature characteristics of a myosin conformation between the pre-power stroke (black), and the rigor-like states (grey). (**A**) The transducer is partially twisted, particularly β-strands 1 to 3. (**B**) The relay helix and the converter approached the rigor-like position. The converter/lever arm must undergo a ~5° rotation to transition to the final rigor down position (grey). The N-terminal domain is markedly shifted towards its rigor position. (**C**) The large actin-binding cleft is found in a position between pre-power stroke (black) and rigor-like states (grey), thereby creating a new actin-binding interface. (**D**) The active site P-loop has moved ~9 Å from its pre-power stroke position in the blebbistatin-bound myosin-II·ADP conformation, but has not reached the rigor position.

Comparison with our apo rigor-like myosin-II and the available pre-power stroke structure (pdb: 1vom) reveals that the major structural features of the blebbistatin-bound myosin-II·ADP conformation are in positions between the pre- and post-stroke states, closer to the final rigor state at the end of the power stroke. In contrast to an earlier proposed conformation of the ternary myosin-II·ADP·blebbistatin complex at the start of the power stroke [37], our structure shows a myosin-II conformation in the presence of blebbistatin and ADP that, according to the characteristic structural elements in the motor domain that were used in the past to define the states of the actomyosin motor cycle, resembles a conformation towards the end of the power stroke, associated with the second converter rotation. The central transducer for chemomechanical coupling in the blebbistatin-bound myosin motor domain is partially twisted, particularly β-strands 1 to 3 (Figure 3A). During the power stroke, the transducer mediates the straightening of the relay helix as well as the repositioning of the N-terminal domain, which allows a continuous interaction of the N-terminal domain and the converter [28]. The N-terminal domain in the myosin-II·ADP·blebbistatin structure is pivoted by ∼2 Å relative to the U50 kDa domain from the corresponding rigor position (Figure 3B). Consistently, the converter is rotated by approximately 55° as compared to the up position in the pre-power stroke state, and would need to undergo only a minor 4° to 5° converter rotation to transition to the down position in the rigor state (Figure 3B). Thus, the rotation amplitude of the converter is more advanced than in the reported strong-Mg^2+^·ADP-bound state (approximately 30° to 50° away from the up position, and 30° to 10° away from the down position) [12,31]. Similarly, a transition by approximately 1 Å of both the inner and outer actin-binding cleft (myosin-II·ADP·blebbistatin complex: inner cleft: 11.7 Å and outer cleft: 11.3 Å) separates the blebbistatin-bound myosin-II·ADP from the fully closed actin-binding cleft as seen in the apo rigor state, indicating a new actin interface (Figure 3C). The status of these important structural elements in the myosin motor domain further suggests that the myosin conformation, as observed in the myosin-II·ADP·blebbistatin complex, might resemble a myosin ADP-release conformation that has already transitioned through the first step of the power stroke and beyond the strong-Mg^2+^·ADP-bound actomyosin state. The conformational changes required to fully close the actin-binding cleft could thus play a role for initiating ADP release from the motor domain.

#### 2.1.2. Structural Features of the Active Site in the Myosin-II∙ADP∙Blebbistatin Complex

In the active site of the blebbistatin-bound myosin-II·ADP structure, considerable rearrangements took place as a consequence of the conformational changes in the actin-binding cleft, the transducer, the N-terminal domain, and the converter. The partial twisting of the transducer induced a shift of switch-1 (^232^RNNNSSR^238^) and switch-2 (^454^DISGFE^459^) by approximately 1 Å towards their rigor positions (Figure 3D), with the critical salt-bridge between Arg238 (switch-1) and Glu459 (switch-2) remaining intact. Recent structural studies supported a P_i_ release mechanism, which presumes the salt-bridge to break in order to open the release tunnel for inorganic phosphate [25]. Our crystal structure features a conformation of the switches in which P_i_ release and actin rebinding appears to have already occurred or is unfeasible. Considering a similar active site in the myosin ADP-release conformation during the power stroke would indicate that the salt-bridge reforms directly after release of P_i_.

**Figure 4:**
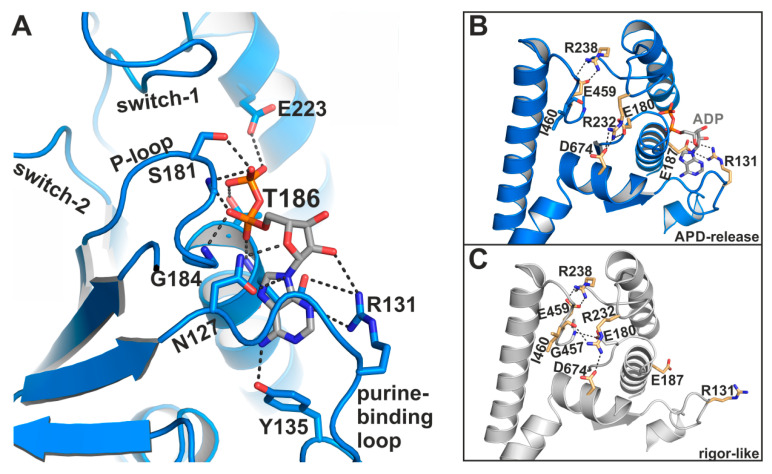
The myosin-II·ADP·blebbistatin crystal structure shows unique conformational features in the active site. (**A**) ADP interacts with its phosphate groups, primarily with the reoriented P-loop. The β-phosphate is partially unbound and oriented towards the surrounding water at the protein surface. The adenosine base of the nucleotide is nestled into a groove, formed by a salt-bridge between Arg131 and Glu187. (**B**) Close-up view on the active site in the myosin-II·ADP·blebbistatin structure shows the critical salt-bridge between switch-1 (Arg238) and switch-2 (Glu459), as well as the newly identified salt-bridge between Arg131 and Glu187 that stabilizes ADP in the release position. A third, complex salt-bridge between Arg232 (switch-1), Asp674 (SH2-helix), and Glu180 (P-loop) seems to be important for mediating the chemomechanical coupling between the active site, the actin-binding region and the mechanical converter/lever arm. (**C**) Close-up view on the active site in the rigor-like, nucleotide-free myosin-II structure. Due to slight adjustments to the active site, the interaction network around Arg232 is altered as compared to the myosin-II·ADP·blebbistatin structure, and Arg232 interacts with Asp674 (SH2-helix), and Ile460 (switch-2), while Glu180 (P-loop) formed a hydrogen-bond to Gly457 (switch-2) in this conformation.

The largest deviations from the active site conformation as seen in the pre-power stroke state were observed for the phosphate-binding P-loop, which moved ~9 Å towards the front entrance of the active site (Figure 3D), accompanied by a rotational movement of the N-terminal domain. Together with the P-loop, the coordinated ADP is repositioned away from switch-1 and -2 towards the protein surface, and is now accessible to the surrounding water in a new binding position. The adenosine base of ADP remains bound to the P-loop through hydrophobic interactions, as well as hydrogen bonds to residues Tyr135 and Asn127 of the purine-binding loop (^126^VNPFKRIPIYT^136^) (Figure 4A). In addition, the sidechain of Arg131 rearranged towards the nucleotide to facilitate the formation of a salt-bridge with Glu187, thereby forming a binding groove for the adenosine base of the nucleotide. This salt-bridge might therefore represent a critical feature for promoting ADP release. Arg131 interacts furthermore with a hydroxyl group of the nucleotide ribose and thereby additionally stabilizes the nucleotide in this new position. While the α-phosphate is involved in a tight interaction network with residues Gly184, Thr186, and Glu187, the nucleotide β-phosphate is orientated away from the P-loop, towards the surrounding water. As a consequence, most of the interactions of the β-phosphate with active site residues that can be observed in known myosin structures are lost in this ADP-bound myosin-II structure (seven interactions in the pre-power stroke state, while three interactions are observed in our structure), suggesting a weakly bound nucleotide with a conformation that is distinct from an earlier structure with ADP soaked into a rigor-like conformation of myosin-V [28]. A reduced number of hydrogen-bonds of the β-phosphate with residues Ser181, Thr186, and Glu223 were detected in the myosin-II·blebbistatin·ADP structure. No electron density was found for the magnesium ion, which usually coordinates the negatively charged phosphates of the nucleotide as well as phosphate-sensor residues Thr186 (P-loop) and Ser237 (switch-1). We assume that due to the movement of the P-loop relative to switch-1, the coordination of Mg^2+^ is disturbed, causing its dissociation from the active site. This is in good agreement with earlier studies, which showed that Mg^2+^ release precedes ADP release for various myosin isoforms [41,42,43].

#### 2.1.3. A Potential Communication Hub Centered on Arg232 Allosterically Mediates Changes in the Active Site to the Converter and Actin-Binding Region

Further stabilizing interactions could be found between the three phosphate-binding loops as a consequence of the rearrangements in the active site. Except for the critical salt-bridge between switch-1 (Arg238) and -2 (Glu459), and the identified salt-bridge between the purine-binding loop (Arg131) and the P-loop (Glu187) that stabilizes the adenosine base of the nucleotide, a third, complex salt-bridge has been formed in the blebbistatin-bound myosin-II·ADP structure between switch-1 (Arg232), the P-loop (Glu180), and the SH2-helix (Asp674) (Figure 4B). This salt-bridge thus seems to couple the active site sensors—switch-1 and P-loop—with the SH2-helix in the N-terminal domain of myosin, indicating a critical role in communicating conformational changes in the myosin motor domain. It is well-known that the different domains and remote sites in myosin are interconnected and highly communicate with each other. This complex salt-bridge might therefore represent an allosteric communication hub for mediating changes in the actin-binding cleft via switch-1 to the active site and the N-terminal domain. The latter in turn needs to positionally dislocate in order to allow the final rotation of the converter/lever arm during the transition to the rigor state. An equivalent complex salt-bridge in scallop myosins has been suggested earlier to control the communication between the actin-binding region and the active site [44].

The corresponding arginine in the rigor-like *Gg* myosin-V [45] structure shows no interactions with the surrounding active site motifs. In our rigor-like apo myosin-II structure, the interactions of Arg232 differ markedly (Figure 4C) from the blebbistatin-bound myosin-II·ADP structure. While Arg232 in the rigor-like state is hydrogen-bonded to Asn674 of the SH2-helix as well, the contact with Glu180 (P-loop) is broken. Instead, Arg232 formed a hydrogen bond with the backbone of Ile460 (switch-2), while Glu180 of switch-1 interacts with Gly457 (switch-2). Hence, both interactions in the rigor-like state assist the maintenance of the closed conformation of the switches, highlighting the central role of the interaction network mediated by Arg232 in chemomechanical coupling in myosin.

### 2.2. Blebbistatin Affects the Myosin Conformation by Binding to the Known Allosteric Binding Pocket

The myosin-II inhibitor blebbistatin has been shown earlier to prevent cleft closure by blocking myosin in the weakly actin-attached pre-power stroke state with the hydrolysis products Mg^2+^·ADP·P_i_ bound to the active site [14,33,34]. This effect gave rise to numerous studies on cytoskeletal and muscle function using blebbistatin as a chemical tool. More recent results suggested blebbistatin to stabilize an additional state at the start of the power stroke with a closed actin-binding cleft but the lever arm was still in the up position [35,36,37]. However, these mechanisms cannot explain all the experimental effects observed with blebbistatin [38], and our high-resolution structure of myosin-II complexed with ADP and blebbistatin resembled a myosin conformation towards the end of the power stroke involved in ADP dissociation from the motor domain.

Blebbistatin binds to the same allosteric binding pocket as reported with the pre-power stroke state myosin-II crystal structure (pdb: 1yv3) [14] at the apex of the large actin-binding cleft and adjacent to the active site phosphate sensors switch-1 and switch-2 (Figure 2C). In agreement with the earlier crystal structures, binding of blebbistatin in the myosin-II·ADP·blebbistatin structure is primarily accomplished via hydrophobic interactions with the U50 linker (the loop following β7 of the transducer), the relay helix, and the W-helix, including residues Tyr261, Ile455, Glu467, Ile471, Thr474, Val630, Tyr634, and Leu641. Particularly amino acids Thr474 and Tyr634 were identified earlier to critically affect the specificity of blebbistatin towards class II myosins [14]. Moreover, as shown before, three hydrogen bonds with residues Gly240, Leu262, and Ser456 contribute to the binding affinity of blebbistatin. The characteristic displacement of the sidechains of residues Leu262 and Tyr634 by approximately 3 to 4 Å is clearly visible also in the blebbistatin-bound myosin-II·ADP structure, which opened the allosteric pocket to accommodate blebbistatin. Hence, the allosteric binding pocket of blebbistatin in our new crystal structure highly resembles the previously observed pocket conformation with conserved protein–ligand interactions, suggesting the presence of the binding pocket in several of the myosin states without larger structural changes of the protein around blebbistatin. In contrast to the postulated inhibitory mechanism of blebbistatin in which switch-2 reorientation is blocked, and thereby P_i_ release and converter rotation, we identified marked positional changes of switch-2 in the presence of ADP and blebbistatin towards its rigor position (Figure 3D). This indicates a much more complex mechanism of blebbistatin that might also affect ADP binding and release.

#### 2.2.1. Blebbistatin Increases the Affinity of Myosin-II for ADP in the Presence and Absence of Mg^2+^

In order to directly analyze the effect of blebbistatin on the bound nucleotide, we performed binding assays using microscale thermophoresis (MST). The sequential release of the hydrolysis products Mg^2+^ and ADP are assumed to drive the conformational changes required for the second step of the mechanical power stroke. Kinetic studies showed that ADP release in distinct myosin-II isoforms is regulated by Mg^2+^ [46], and for myosin-V and presumably various other myosin classes, both the myosin·Mg^2+^·ADP and myosin·ADP states exist in equilibrium, facilitating dissociation of Mg^2+^ to precede ADP release at physiological magnesium concentrations [41,43]. In accordance with this, and assuming our crystallized structure resembles the ADP-release conformation during the end of the myosin power stroke, we did not find electron density for the Mg^2+^ ion in the myosin-II·ADP·blebbistatin structure.

**Figure 5:**
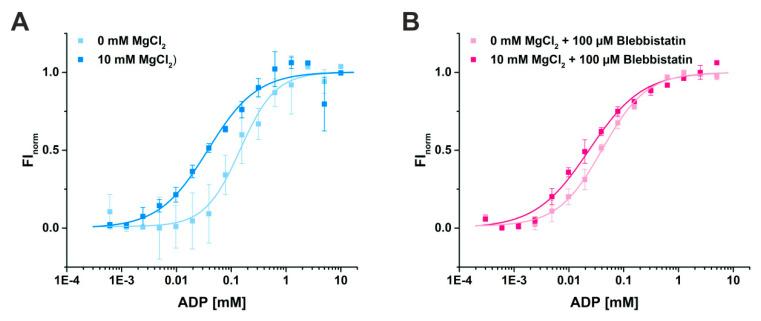
Binding assays of ADP to myosin-II using microscale thermophoresis. (**A**) The affinity of ADP for myosin-II in the absence of magnesium is reduced from K_D_ = 37.5 ± 7.3 µM (10 mM Mg^2+^) to 145.8 ± 19.3 µM (0 mM Mg^2+^). (**B**) Addition of 100 µM blebbistatin increased the binding affinity of ADP for myosin-II, both in the presence (K_D_ = 21.2 ± 3.4 µM) and absence of magnesium (K_D_ = 40.6 ± 3.2 µM).

Using MST, we determined a binding constant (K_D_) for ADP of 37.5 ± 7.3 µM in the presence of 10 mM Mg^2+^ (Figure 5A). As expected, the binding affinity of ADP to myosin-II was decreased by a factor of 4 to a K_D_ of 145.8 ± 19.3 µM in the absence of magnesium, consistent with a sequential release mechanism during force generation with Mg^2+^ released through an as yet unknown route. Addition of blebbistatin slightly increased the binding affinity of ADP for myosin 1.8-fold to a K_D_ value of 21.2 ± 3.4 µM in the presence of 10 mM Mg^2+^ (Figure 5B), which is in good agreement with previous measurements of the ADP affinity to the actomyosin complex in the presence of Mg^2+^ and blebbistatin that reported a K_D_ value of 24 ± 1 µM [37]. Interestingly, without Mg^2+^ present, the affinity of ADP for myosin-II (K_D_ of 40.6 ± 3.2 µM) in the presence of blebbistatin is significantly elevated up to a factor of 4 as compared to the K_D_ value in the absence of the small molecule inhibitor (Figure 5B). These results correlate well with our new myosin-II·ADP·blebbistatin structure, and we therefore speculate that blebbistatin stabilizes the crystallographically found ADP-release-like conformation of myosin-II through an increased ADP affinity while Mg^2+^ is already dissociated from the myosin.

#### 2.2.2. Blebbistatin Stabilizes the Crystallographically Observed Myosin-II∙ADP Conformation

**Figure 6:**
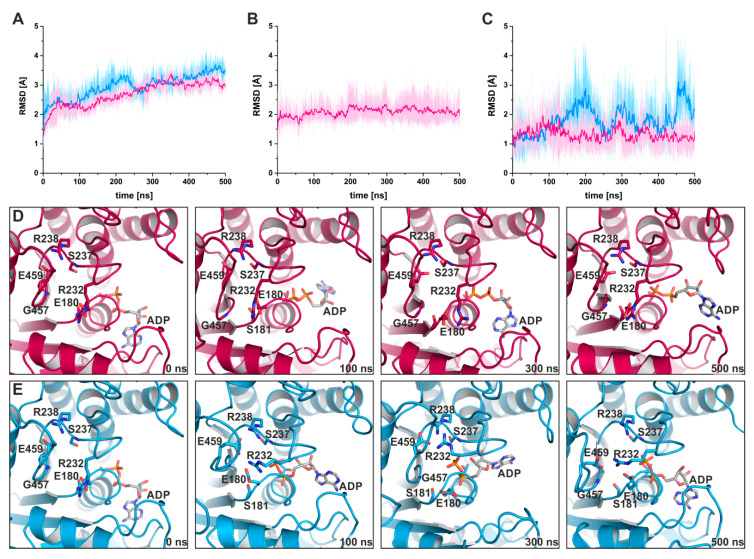
Classical molecular dynamics (cMD) simulations support the stabilizing effect of blebbistatin on the crystallographically determined conformation of myosin-II. (**A**) Root mean square deviations of the protein backbone atoms, averaged over duplicate 500 ns cMD simulations of blebbistatin-bound (magenta) and -unbound (blue) myosin, confirm the stabilizing effect of blebbistatin on the crystallized conformation, while in the absence of blebbistatin, larger deviations from the start structure were observed. (**B**) Blebbistatin remains embedded in its binding pocket throughout the simulations, showing only negligible deviations from its crystallized binding pose. (**C**) Root mean square deviations of the converter indicate no movement of the converter while blebbistatin is bound (magenta); however, a smaller shift towards the pre-power stroke position of the converter is monitored in the absence of blebbistatin (blue). Shown in diagrams A-C are the averages of duplicate cMD simulations. (**D**) The positions of active site phosphate sensors are stabilized spatiotemporally along the cMD simulations in the presence of blebbistatin, with the critical salt-bridge formed. Binding of ADP in the crystallized conformation appeared loose during the cMD simulations with considerable fluctuations of the nucleotide. (**E**) During cMD simulations in the absence of blebbistatin, the P-loop moved towards switch-1 and -2 in the reversed direction of the power stroke. The nucleotide ADP was markedly shifted together with the P-loop inside into the active site.

To further support this hypothesis, we carried out 500 ns classical molecular dynamics simulations (cMD) in duplicates with a total of more than 2 µs simulations in explicit solvent and in the absence and presence of blebbistatin. Starting from the blebbistatin-bound myosin-II·ADP crystal structure, we did not observe any larger conformational changes of the myosin motor domain along the cMD trajectories with mean rmsd values of the protein backbone around 3 Å (Figure 6A). A minimal shift of blebbistatin within the allosteric pocket towards the relay helix occurred, while remaining bound to the relay helix, the W-helix, and the transducer through the crystallographically determined interactions (Figure 6B). No significant rearrangements of the relay helix together with the converter were detected along the cMD simulations (Figure 6C), and the converter stably interacted with the N-terminal domain throughout the simulations. The active site switches as well as the P-loop showed only negligible fluctuations and remained in their unique crystallographically determined positions in the blebbistatin-bound myosin simulations (Figure 6D). These positions of the switches were spatially restrained by the stable critical salt-bridge between Arg238 (switch-1) and Glu459 (switch-2) along the trajectories. However, the bound ADP in its new position at the surface along the outwards shifted P-loop showed substantial fluctuations around its crystallographic position with an average rmsd of 5.4 Å. Along the trajectories, ADP seems therefore loosely coupled to the P-loop with a number of transient interactions, indicating a weak ADP association. These results demonstrate that blebbistatin is capable of stabilizing a unique conformation of myosin-II with a rearranged P-loop towards the front entrance of the active site and a loosely bound ADP.

In contrast, during the cMD simulations in the absence of blebbistatin while starting from the same crystal structure, the bound ADP together with the P-loop moved linearly backwards in the direction of their pre-power stroke state positions (Figure 6E), dragging the nucleotide ~5 Å inside the active site. Concomitantly, a slight untwisting of the transducer could be observed along the trajectories, which led to the decoupling of switch-1 and switch-2 by breaking the critical salt-bridge, and pronounced rearrangements of the switches. The entire protein appears to be subject to a partial reverse motion of the power stroke, particularly of the N-terminal domain and the adjacent converter domain, which drew closer to their pre-power stroke state positions. As a consequence of the loss of the critical salt-bridge at a relatively early timestep of the cMD simulations, switch-1 forms a new complex salt-bridge via residue Arg232 with Glu459 (switch-2) and Glu180 (P-loop), highlighting the critical role of Arg232 as a fulcrum for mediating conformational changes between remote sites in the myosin motor domain. In addition, Gly457 of switch-2, usually involved in binding the γ-phosphate of ATP or the cleaved P_i_, interacts with amino acid Gly179 of the P-loop. Inside the active site, ADP rebinds to the main chain atoms of residue Ser237 (switch-1), a critical amino acid that typically coordinates the Mg^2+^ ion in the active site, which in turn interacts with the β-phosphate of the nucleotide. Hence, in the absence of blebbistatin, the nucleotide seems to bind more deeply inside the myosin active site, and myosin-II at the end of our 500 ns cMD simulations appears to adopt a conformation resembling the strong-ADP-bound myosin state, determined by cryoEM [12,31].

Comparing our results with these strong-ADP cryoEM models demonstrated that myosin in this conformation would require only small rearrangements of the N-terminal domain together with the relay helix and the converter by approximately 10° to reach the final rigor state. Moreover, a similar unique coordination of ADP by the active site switch motifs was postulated earlier [31] with directly coupled switch-1 and P-loop, as observed in our myosin-II·ADP·blebbistatin crystal structure; however, the resolution limit of the cryoEM model in that study prevented a direct comparison of the sidechain and nucleotide positions, and thus the final validation that the conformation at the end of our simulations represents the strong-ADP-bound myosin state. Nevertheless, our cMD simulations suggest that the conformation of myosin-II, stabilized by blebbistatin, can reversibly adopt a conformation, similar to the strong-Mg^2+^·ADP state in the absence of blebbistatin, and might therefore resemble a conformation towards the end of the power stroke with the ADP loosely bound and involved in ADP dissociation from the motor domain.

### 2.3. The ADP Release Pathway Reveals a Central Role of Arg131 for Guiding ADP Dissociation

#### 2.3.1. Computational Generation of a Strong-ADP-Bound Myosin-II Conformation, and First Step of the Power Stroke

The positions of critical structural elements in our crystal structure of myosin-II in complex with ADP and blebbistatin suggest a resemblance with a myosin conformation towards the end of the power stroke that is involved in ADP release. To gain further insights into the molecular events underlying the mechanism of the second step of the myosin-II power stroke associated with ADP release, we used a set of unbiased and pulling molecular dynamics simulation techniques.

During the power stroke, the population of a myosin state with strongly bound Mg^2+^·ADP after rebinding of the myosin motor to its actin filament track and P_i_ release was shown in recent cryoEM studies of myosin-I, -V, and -VI [12,13,31]. We therefore computationally generated a myosin-II conformation, resembling this strong-ADP-bound myosin state by applying an external biasing force to the actin-binding cleft and switch-2 of the active site, in order to force cleft closure and twisting of the β-strands of the central transducer, similar to what had been published earlier for myosin-II [24]. This external force mimics the effect exerted by the actin filament during rebinding of the myosin motor domain. The final model of these targeted molecular dynamics (TMD) simulations possessed the same critical features as the reported strong-ADP state, including a closed actin-binding cleft, and a partially twisted transducer, which in turn initiated an approximately ⅓ rotation of the converter towards the rigor down position. Mg^2+^·ADP remained coordinated in the active site by switch-1, switch-2, and the P-loop.

#### 2.3.2. Computational Analysis of the Second Step of the Power Stroke

**Figure 7:**
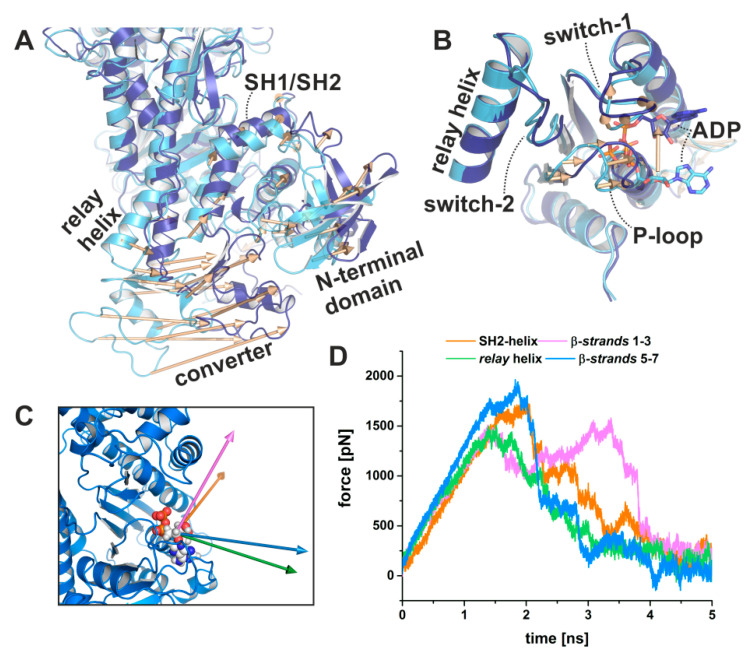
Pulling MD simulations using biasing forces on the converter domain visualize coupled conformational transitions in myosin and ADP dissociation. (**A**) Targeted molecular dynamics (TMD) simulations of the converter rotation from the up position of a strong-ADP-bound myosin conformation with closed actin-binding cleft and partially twisted transducer (light blue) towards the down position (dark blue) were coupled to rearrangements of the N-terminal domain. Structural changes are indicated by vectors. (**B**) The induced converter rotation during the TMD simulations led to a significant ~6 Å shift of the P-loop towards the front entrance of the active site. The bound ADP moved together with the P-loop towards the surface and lost interactions, which finally led to larger fluctuation of the nucleotide, and the population of a conformation similar to the myosin-II·ADP·blebbistatin crystal structure. (**C**) Visualization of the release vectors used to pull the nucleotide out of the crystallized position in the myosin motor domain. (**D**) Pulling forces required to dissociate ADP from myosin along the four different vectors. Independent of the release vector, a consistent set of interactions was involved in ADP dissociation. Shown are the averages of duplicate steered MD simulations.

Starting from this intermediate strong-ADP-bound myosin conformation, we used subsequent TMD simulations with pulling forces applied to the converter in order to drive the domain towards the down position as observed in the rigor structure (Figure 7A), while at the same time positionally restraining the actin-binding cleft to maintain a closed conformation. Hence, our simulations mimic the converter rotation in an actin attached state during the power stroke, while the major part of the myosin motor remains unbiased, particularly the active site, the N-terminal domain, and the relay helix. Accompanying this second rotation of the converter/lever arm, the P-loop moved towards the front entrance of the active site by up to ~6 Å (Figure 7B), pulling the nucleotide out of its pre-power stroke binding position. Neither the converter, nor the P-loop, however, reached the complete rigor position within 5 or 10 ns TMD simulations. At the end of the simulations, the converter showed a straightened relay helix and a maximal rotation of ~55.5° (Figure 7A), which is in good agreement with the conformation observed in our crystal structure in the presence of blebbistatin and ADP. The critical salt-bridge between switch-1 and switch-2 stayed intact over the TMD trajectories, with Glu459 interacting transiently with Arg232 in one simulation replica.

Moreover, our simulations further demonstrated the role of Arg232 in communicating changes of the active site to other regions in the myosin motor domain. The arginine residue Arg232 was hydrogen-bonded throughout the simulations to Glu180 of the P-loop. In addition to the transient interaction with the salt-bridge residue Glu459 of switch-2, Arg232 formed a stable interaction with the main chain of Gly457 (switch-2), which is broken after approximately 4.5 ns TMD simulation time. Concurrently, the P-loop seemed to have reached a critical distance to the switches, and Arg232 lost its contacts to switch-2 by following the movement of the P-loop via a strong interaction with Glu180 (P-loop). Residue Asp674 of the SH1/SH2-region in the N-terminal domain drew closer towards Arg232 along the trajectories, but remained outside the distance for an attractive interaction with the arginine residue. Hence, the information of the initial converter rotation might be communicated through a different pathway, without the involvement of Arg232.

An average of approximately six hydrogen bonds were detected between ADP and the protein over the trajectories and allowed the nucleotide to follow the unbiased, almost linear movement of the P-loop towards the surface. In particular, interactions of P-loop and switch-1 residues stabilized ADP binding during the active site rearrangements with highly populated hydrogen bonds between ADP and residues Lys185 (occupancy: 180.0%), Ser237 (occupancy: 67.6%), Ser181 (occupancy: 60.4%), Gly182 (occupancy: 58.0%), Asn235 (occupancy: 37.2%), Asn127 (occupancy: 36.4%), Asn233 (occupancy: 35.0%), Thr186 (occupancy: 29.4%), and Gly184 (occupancy: 27.2%). After ~2 ns TMD simulation time, binding of the ADP phosphates appeared to be loosened, indicated by increased fluctuations of the nucleotide (Figure 7B). The ADP thus explored an increased conformational space, which is in good agreement with a previous crystal structure of scallop myosin, showing ADP in an unusual, partially unbound position in the active site [44]. The salt-bridge between Arg131 and Glu187 intermediately formed and seemed to have a stabilizing function during the conformational transitions in the active site. We hypothesize that the Arg131-Glu187 salt-bridge is important for the myosin to stabilize the bound ADP, which is also supported by our crystal structure that showed a new binding groove for the adenosine base, formed by the interaction between Arg131 and Glu187. Accordingly, breaking of this salt-bridge during the TMD simulations was directly linked to increased fluctuations of the nucleotide and particularly the adenosine base. Our computational analysis therefore underpins an intimate coupling between the start of the second convert rotation of the power stroke with rearrangements of the active site, particularly the P-loop, mediated by the twisting motion of the transducer, and is consistent with the power stroke mechanism discussed previously involving the strong-Mg^2+^·ADP-bound state [12,31]. In addition, our simulations indicate that myosin alone, in the absence of the inhibitor blebbistatin, can adopt a conformation during the second converter rotation that shows high similarities with the myosin conformation in our crystallized myosin-II·ADP·blebbistatin complex, with all critical structural elements, involved in chemomechanical coupling, in comparable positions. This is in good agreement with various earlier studies, which showed that other known myosin modulators trap the myosin motor in distinct conformational states of the motor cycle, inducing only smaller local structural changes [14,15,16,17]. The applied simulations were performed in the absence of blebbistatin and were unbiased in the way that the myosin-II·ADP·blebbistatin crystal structure was not included during our TMD simulations, but we induced the power stroke by forcing the distant converter to rotate, which eventually led to associated changes in the motor domain and particularly the active site, thereby populating a conformation that resembled our blebbistatin-bound myosin-II structure.

#### 2.3.3. ADP Release from Myosin as Analyzed by Steered Molecular Dynamics Simulations

The second rotation of the converter during force generation, however, is assumed to involve additionally ADP dissociation from the myosin-II motor domain. We used a series of steered molecular dynamics (SMD) simulations in order to determine the potential release pathway of ADP from the myosin active site, as observed in the blebbistatin-bound myosin-II·ADP structure and considering the position of ADP resembles the ADP-release state of myosin, as well as the associated conformational changes and specific interactions during the transition. Various escape routes for ADP were explored by applying a pulling force on the center of mass of the nucleotide along four different release vectors from the ADP position, as found in the crystal structure (Figure 7C). Strikingly, our SMD results suggest that independent of the chosen release vector, the primary interacting residues along the SMD trajectories, and the pulling forces required for dissociating ADP from the myosin motor domain were highly consistent between the different SMD simulations (Figure 7D), suggesting a divergent dissociation pathway potentially dominated by thermal fluctuations.

A maximum force of up to ~2000 pN was required, associated mainly with the breaking of the hydrogen bonds between the ADP phosphates and the P-loop residues Ser181, Thr186 and Glu187. Other important interactions during ADP dissociation along the SMD simulations included Tyr135 (purine-binding loop), Arg131 (purine-binding loop), Asn127 (purine-binding loop), Ala183 (P-loop), Gly184 (P-loop), Lys185 (P-loop), Gly182 (P-loop), Thr186 (P-loop), Asn235 (switch-1), and Lys190 (F-helix). Most of these interactions were observed also in the myosin-II·ADP·blebbistatin crystal structure to play a role in coordinating ADP in the partially unbound conformation. A generally lower pulling force was necessary to disrupt the interactions with the adenosine base, which might correlate with the increased fluctuations of the ADP adenosine base and a loosened interaction. Although the sequence of bond breaking was dependent on the chosen vector, the SMD simulations highlighted the strong interaction of the β-phosphate to the sidechain of residue Thr186, together with a role of Arg131 in guiding ADP from the myosin motor domain. The majority of obtained SMD trajectories showed Arg131 of the purine-binding loop to interact with the ADP either by stacking interactions with the adenosine base or by hydrogen bonds to the phosphates.

**Figure 8:**
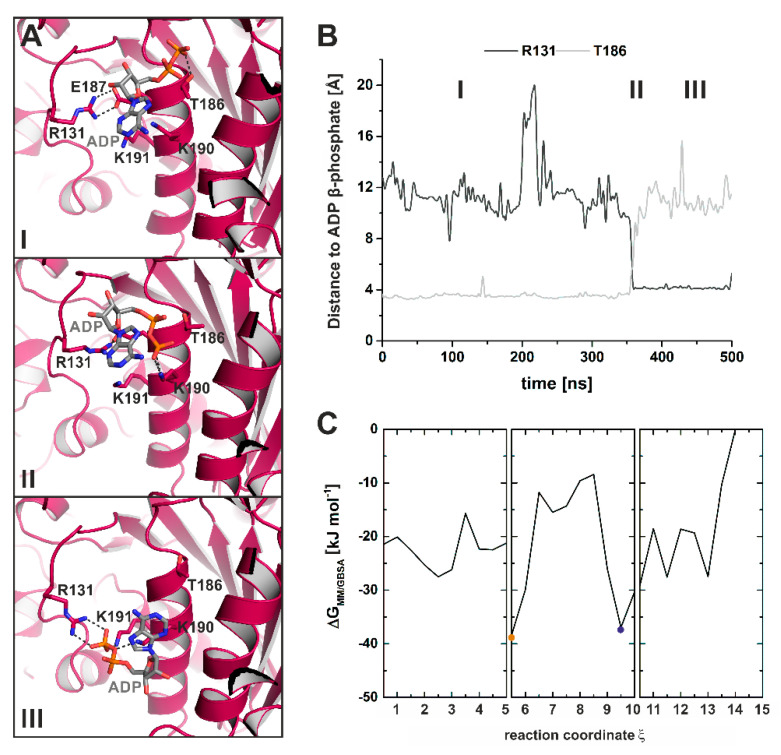
Unbiased MD simulations captured a potential ADP release pathway. (**A**) Molecular events during ADP dissociation along the unbiased MD trajectories reveal a guiding mechanism of Arg131 together with Thr186. Panels I through III show snapshots along the cMD trajectory. (**B**) Distance of the protein residues Arg131 and Thr186 to the nucleotide β-phosphate over the MD simulations illustrate the interplay between the two protein residues in the proposed ADP release mechanism. (**C**) Energy profile of the reconstructed ADP release pathway from a strongly bound ADP to nucleotide dissociation as determined by the molecular mechanics/generalized-born surface area (MM/GBSA) interaction energy between the protein and ADP, obtained by combining TMD and cMD trajectories. Both the myosin conformation with strongly bound ADP (orange dot) and the myosin conformation resembling our myosin-II·ADP·blebbistatin structure (blue dot) represent energy minima (low-energy conformations) along the reconstructed myosin power stroke.

Further evidence of the function of Arg131 in guiding the release of ADP was obtained using unbiased MD simulations (classical MD simulations) (Figure 8A,B). In these cMD simulations, dissociation of ADP started with increased fluctuations of the nucleotide around its new position at the surface of the active site, thereby exploring a larger conformational space, including conformations with transient interactions to the loop-1-flanking F- and G-helices. These spatiotemporal changes in the ADP binding position led to an initial dissociation of the phosphate groups from the P-loop. Stable interactions of the β-phosphate were still found with residues Ser181 and Thr186 (Figure 8A(I),B), which is in agreement with our SMD simulations. Simultaneously, the salt-bridge between Arg131 and Glu187 was broken. In our crystal structure, this salt-bridge was identified to stabilize the adenosine base of the nucleotide in the new ADP position. Subsequently, the hydrogen bonds between Ser181/Thr186 and the β-phosphate were sequentially disrupted, and the nucleotide was shifted towards Arg131 by Lys190 (Figure 8A(II)). Arg131 coordinated ADP together with Lys190 and Lys191, and guided the nucleotide further towards the surface and the surrounding water (Figure 8A(III)). This is in agreement with a scallop myosin crystal structure in complex with a partially unbound ADP [44], and demonstrates that Arg131 might play a crucial role for the ADP release mechanism by taking over ADP from Thr186 and guiding the nucleotide out of the active site.

Combining our TMD and unbiased cMD simulations allowed us to reconstruct a potential ADP release pathway from the myosin state with strong ADP binding in the active site to the dissociation of the nucleotide from the myosin motor domain, and to monitor the associated structural changes during the transitions. Computing an energy profile along the obtained release pathway by means of the interaction energy (ΔG_MM/GBSA_) between the protein and ADP using the molecular mechanics/generalized-born surface area (MM/GBSA) method further illustrates that both the strong-Mg^2+^·ADP-bound state and the conformation, resembling our crystallized myosin-II structure in complex with ADP and blebbistatin, represent energy minima, suggesting low-energy conformations along the reconstructed power stroke (Figure 8C). During the transition from the initial binding position in the active site with strong interactions to the switch motifs towards the ADP position as observed in our crystal structure, the nucleotide traversed a number of energy barriers, with reduced ΔG_MM/GBSA_ and therefore ADP affinity to the protein up to ΔΔG_MM/GBSA_ of ~30 kJ·mol^-1^. These transiently decreased ADP affinities are mostly related to positional shifts of the nucleotide towards switch-1 and the G-helix, often linked to an intermediate breaking of the nucleotide-stabilizing salt-bridge between Arg131 and Glu187. Moreover, the combined simulations indicated a late formation of the attractive interactions between Arg232 and the SH2-helix in the N-terminal domain via Asp674, suggesting a role during the final adjustments in myosin prior to ADP dissociation in order to eventually drive the converter to the rigor down position.

## 3. Discussion

Myosin is a molecular motor cycling through a series of complex conformational transitions while transforming chemical energy into mechanical work. The structural characterization of the relevant states and conformational transitions between them at the atomic level is crucial for our understanding of the underlying molecular mechanism of force production. The release of ADP from myosin motors is a critical process in the actomyosin motor cycle and occurs following the rearrangement of the active site P-loop towards the surface. It has been shown that load can prolong the duty ratio of myosin motors, which is the time the motor stays strongly attached to actin filaments, by affecting the conformational transitions, required for nucleotide release, and thereby the rate of ADP dissociation [47,48,49]. Hence, release of ADP represents a regulatory mechanism for the actomyosin motor cycle to control the mechanical output and type of motility. According to the proposed two-step mechanism for force production during the myosin power stroke [12,13,31], ATP hydrolysis induces rebinding of the myosin motor to the actin filament and the release of the hydrolysis product P_i_, driving the motor to a strongly actin-attached conformational state with tightly bound Mg^2+^·ADP. The transition to this strong-Mg^2+^·ADP-bound state comprises the major, first rotation of the converter and adjacent lever arm [12,13,31]. A second rotation (second step of the power stroke) is linked to the release of the hydrolysis products Mg^2+^ and ADP.

In this work, we report the structural characterization of a new myosin-II conformation that was obtained in the presence of ADP and the small molecule inhibitor blebbistatin. The overall conformation of our high-resolution myosin-II crystal structure and specifically the characteristic structural elements, which were used previously to assign new structures as potential states of the actomyosin cycle, are found in unique positions between the myosin-II pre-power stroke state and the rigor state at the end of the power stroke, including the large actin-binding cleft between subdomains U50 and L50 kDa, the central transducer, the relay helix, and the mechanical converter/lever arm. In particular, the converter is found in a position where it must undergo only an approximately 5° rotation to its rigor position. Thus, the converter in our crystal structure is further rotated than the converter in the previously published strong-Mg^2+^·ADP state (approximately 10° rotation required to reach the rigor position). The large observed rearrangement of the active site P-loop by approximately 9 Å towards the surface, which relocated the bound ADP out of its active site binding position towards the front entrance in a partially unbound position, suggests a weakened binding of ADP. This myosin conformation exhibits structural features which are markedly different from earlier structures. The specific binding pose of ADP might promote nucleotide dissociation and differs clearly from the previous scallop myosin structure with a dislocated, partially unbound ADP inside the active site [44]. All critical structural elements in the active site of our crystal structure are in positions that seem to precede the rigor positions as well as the rigor-like conformations of a myosin-V structure with soaked ADP [28]. The absence of Mg^2+^ in our ADP-release structure supports the postulated sequential release of Mg^2+^ and ADP from the myosin motor domain [41,42,43].

In conclusion, the overall conformation and particularly the positions of the characteristic structural elements involved in chemomechanical coupling suggest that our crystallized myosin-II·ADP·blebbistatin structure resembles a myosin conformation towards the end of the power stroke, involved in the second step of converter/lever arm swing during ADP release, following the strong-Mg^2+^·ADP-bound myosin state. Although we cannot fully exclude on the basis of our data the possibility that the conformation in our crystal structure is only a consequence of the bound inhibitor blebbistatin, and the ADP-release conformation of myosin-II is significantly different, previous results with other small molecule myosin modulators [14,15,16,17], which trapped myosin in distinct states along the actomyosin motor cycle, and specifically the positions of the critical structural elements of the motor strongly foster our hypothesis that the crystallized myosin-II structure complexed with ADP and blebbistatin is very similar to the native myosin ADP-release conformation. Considering that the structure represents the ADP-release conformation, we postulate that the transition from the strong-Mg^2+^·ADP-bound myosin state as observed with cryoEM [12,13,31] to the ADP-release conformation might be driven by the release of Mg^2+^, which leads to repositioning of the switches in the active site, coupled to the initiation of the second converter/lever arm rotation. This transition in turn decreases the affinity of ADP, which is underpinned by accompanying binding assays in the absence of Mg^2+^, and might prepare myosin for ADP release and the final adjustment of the converter towards the rigor down position.

Computational and energetic reconstruction of the ADP release pathway from the crystallographically resolved pre-power stroke state (with a strongly bound ADP) up to nucleotide dissociation from the motor domain suggests the population of low-energy conformations along the release pathway that highly resemble both the proposed strong-Mg^2+^·ADP-bound state and the myosin conformation as seen in our blebbistatin-bound myosin-II·ADP crystal structure, without including the two experimental structures in the computations a priori. Hence, myosin alone seems to be able to adopt a similar conformation as our crystallized inhibitor-bound myosin structure, which is in good agreement with studies on various small molecule myosin inhibitors [14,15,17] and activators [16] that trap myosin in specific conformations that can be assigned as structural states of the actomyosin motor cycle. These results further corroborate the hypothesis that the overall crystallized structure in the presence of blebbistatin resembles a native myosin-II conformation along the power stroke.

A salt-bridge between residues Arg131 and Glu187 seems to play a critical role in stabilizing the adenosine base of ADP during the reconstructed transition and was found to form a transient binding groove for the nucleotide base in the myosin-II·ADP·blebbistatin structure. In addition, we identified a tight chemomechanical coupling between the converter rotation and the rearrangement of the active site P-loop, mediated by a communication hub centered on residue Arg232. This arginine residue interlinked important structural elements through a complex interaction network, including switch-1, switch-2, and the P-loop of the active site, as well as the SH1/SH2-region in the N-terminal domain, thereby facilitating allosteric communication of the active site with the remote actin-binding cleft and the mechanical converter/lever arm. Arg232 has been discussed earlier to play a critical role for myosin function through a complex salt-bridge found in a scallop myosin crystal structure [44], and this is consistent with studies suggesting particular arginine residues to serve as key connectors between functional subunits in proteins [50]. In conclusion, our structural and computational study sheds light on a new myosin conformation and the two-step mechanism of the force-generating myosin power stroke, providing molecular insights into the potential conformational transitions associated with ADP release, thereby contributing to recent progress [24,25,26,31,51] in resolving the mechanism of force production and cellular motility by molecular motors.

## 4. Materials and Methods

### 4.1. Protein Preparation

The His_6_-tagged motor domain construct of *Dd* myosin-II comprising amino acids 1–765 fused to a C-terminal SSB tag [52,53] was transformed into AX3-Orf^+^ cells by electroporation. Cells were grown in HL5C medium containing 10 µg/mL G418 and 20 U/mL penicillin/streptomycin at 22 °C, subsequently harvested by centrifugation, and resuspended in Buffer A (50 mM Tris-HCl, 2 mM EDTA, 0.2 mM EGTA, 1 mM DTT, 5 mM Benzamidine, 0.1 mM PMSF), pH 8.0). The sonified cell lysate was mixed with Buffer B (Buffer A + 1% Triton X-100, 100 U CIAP) and incubated for 1 h at 4 °C. After centrifugation at 26000 g twice for 1 h the pellet was homogenized in Buffer C (50 mM HEPES, 30 mM KAc, 15 mM Mg(Ac)_2_, 3 mM Benzamidin, 7 mM 2-Mercaptoethanol, 0.1 mM PMSF, pH 7.3) containing 15 mM ATP and 150 mM KCl, and finally centrifuged at 260000 g for 1 h. The supernatant was applied to a Ni-NTA agarose column, pre-equilibrated with Buffer D (50 mM HEPES, 100 mM KCl, 3 mM Benzamidin, pH 7.3), washed with Buffer D, Buffer E (Buffer D including 300 mM KCl), 5% Buffer F (Buffer D + 500 mM imidazole) and finally eluted using a linear gradient from 50 to 500 mM imidazole. Fractions containing recombinant myosin were pooled, dialyzed against Buffer G (50 mM Tris-HCl, 0.5 mM EDTA, 0.2 mM EGTA, 150 mM KCl, 1 mM Mg(Ac)_2_, 1 mM DTT, pH 8.0) and further purified by size exclusion chromatography. The myosin protein was concentrated and stored in Buffer G containing 3% sucrose at −80 °C.

### 4.2. Crystallization, Data Collection and Procession

Crystals of the protein–ligand complex were obtained by co-crystallizing the *Dd* myosin-II construct with 0.5 mM blebbistatin, 2 mM ADP, and 2 mM MgCl_2_ at 4 °C using the sitting drop vapor diffusion method and micro seeding. The mixture was preincubated for 20 min and subsequently mixed with an equal volume of reservoir containing 0.2 M sodium fluoride and 21% PEG 3350. Crystals were cryoprotected with ethylene glycol prior to data collection at synchrotron beamline Proxima-2A at SOLEIL (St. Aubin, France). Crystals of *Dd* myosin-II in the absence of nucleotide were obtained using micro seeding at 4 °C and equal volumes of reservoir containing 0.1 M HEPES pH 7.5, 10% PEG 8000 and 8% ethylene glycol. Crystals were cryoprotected with glycerol prior to data collection at beamline Proxima-2A at SOLEIL (St. Aubin, France).

The datasets were processed with XDS [54] and scaled with AIMLESS [55] from the ccp4 software suite [56]. Molecular replacement using the *Dd* Myosin-II rigor-like structure (pdb: 2aka) [36] as starting model was carried out with phaser [57]. Final model building and structure refinement was performed using Coot [58] and phenix.refine [59]. The final model and structure factor amplitudes were deposited in the Protein Data Bank (www.rcsb.org) [60] with accession codes 6Z7T and 6Z7U. Refinement statistics are listed in Table 1.

### 4.3. Classical Molecular Dynamic Simulations

Classical molecular dynamics (cMD) simulations were performed using NAMD 2.13 [61] and the CHARMM36 force field [62]. Parameters for blebbistatin were generated using the CHARMM General Force Field [63]. The myosin was solvated with the TIP3P explicit water model [64], and neutralized by addition of sodium counter ions. A minimum distance of 9 Å between the protein and water box edges was used with periodic boundary conditions for all simulations. Langevin dynamics and the Langevin piston method maintained a constant temperature of 310 K and a constant pressure of 1 atm. Van der Waals and short-range electrostatic interactions were cut at 12 Å and the particle-mesh Ewald method [65] was used for long-range electrostatic interactions. The solvated systems were initially energy-minimized and equilibrated for at least 5 ns, before the production runs. All cMD simulations were conducted in duplicates for 500 ns with an integration timestep of 2 fs using the resources of the North-German Supercomputing Alliance (HLRN). Simulation trajectory analysis was performed using VMD 1.9 [66].

### 4.4. Targeted Molecular Dynamic Simulations

Targeted molecular dynamics (TMD) simulations were used to induce conformational changes of the proteins in a controlled manner by applying an external force on defined atoms of a starting structure, thereby directly visualizing movements of distinct subdomains in the biomolecules with timescales often inaccessible to cMD simulations [67]. The force applied to each TMD atom was calculated via an additional energy term:

(1) $$V_{TMD}=\frac{1}{2}\frac{k}{N}{\left[RMSD\left(t\right)-RMSD^{*}\left(t\right)\right]}^{2}$$

For TMD simulations, the solvated systems were initially energy-minimized, and subsequently the solvent was equilibrated for 200 ps with restraints on the protein atoms, before the entire system was equilibrated for another 100 ps. Five nanosecond TMD runs were conducted to close the actin binding site and reach a strong-ADP-bound state with an integration time step of 1 fs. A force constant of *k* = 200 kcal mol^-1^ Å^-2^ was applied to the backbone atoms of residues 338–355, 373–382, 386–394, 411–441, 510–518, 525–534, and 540–551 (number of TMD atoms *N* = 388). *RMSD(t)* represents the current root mean square deviation value of the coordinates from the target structure at time *t* and RMSD^*^(t) is the default value at time t, linearly decreasing from the starting structure to the target structure. After closure of the cleft, the converter rotation was initiated in a second 5 ns TMD simulation with a force constant of *k* = 200 kcal mol^-1^ Å^-2^ on the backbone atoms of residues 116 to 119, 122 to 126, 173 to 178, 239 to 247, 253 to 261, 649 to 656, 693 to 697, 736 to 739, and 743 to 749 (number of TMD atoms *N* = 256), while constraining the actin-binding cleft in a closed conformation. All TMD simulations were performed in duplicates.

### 4.5. Steered Molecular Dynamic Simulations

Steered molecular dynamics (SMD) simulations [68,69] were used to analyze the unbinding pathways of the nucleotide from the myosin motor domain by applying an external force on the nucleotide. For the SMD simulations, the solvated and neutralized systems with a minimum distance of 15 Å between the protein and water box edges were initially energy-minimized and stepwise equilibrated as described above. The center of mass of the nucleotide ADP was pulled with a constant velocity of 0.005 Å ps^−1^ along a predefined reaction coordinate ξ with a harmonic force constant of 4 kcal mol^−1^ Å^−2^. The SMD simulations were carried out for 5 ns with an integration timestep of 1 fs in duplicates. To avoid drifting of the systems, positional restraints were applied to the different sets of backbone atoms: either residues 116 to 126 and 649 to 656 or residues 670 to 678 or residues 240 to 249 and 254 to 261 and 447 to 453 or residues 472 to 479.

### 4.6. Microscale Thermophoresis

Microscale thermophoresis (MST) [70,71] was used to measure the affinity of ADP to myosin in the presence or absence of magnesium chloride as well as with and without 100 µM Blebbistatin. Purified *Dd* myosin-II was labeled with atto-647 maleimide dye (Atto-Tec, Siegen, Germany), which forms a chemically stable thio-ether bond with cysteines in the protein. Labeling was performed in MST-buffer (20 mM HEPES, 100 mM NaCl, pH 7.3) for 30 min at room temperature. After buffer exchange with MST-buffer + 0.5 mg/mL BSA, 0.05% tween-20 and either 10 mM MgCl_2_ or 5 mM EDTA, the protein (with a final concentration of 10 nM) was mixed with a 1:1 dilution series of ADP (highest concentration: 10 mM). Experiments were conducted using the Monolith NT.115Pico (Nanotemper, Munich, Germany) at 5% LED power and 40% MST power. The dissociation constant *K_D_* was calculated by plotting the normalized data against the total ADP concentration and fitting with the Hill equation.

## Figures and Tables

**Table 1:** Data collection and refinement statistics.

	*Dd* Myosion-II (apo)	*Dd* Myosin-II·ADP·Blebbistatin
**Data collection**		
Space group	P12_1_1	P2_1_2_1_2_1_
Cell dimensions		
*a*, *b*, *c* (Å)	56.68, 174.40, 100.25	47.37, 88.70, 199.89
α, β, γ (°)	90.00, 106.38, 90.00	90.00, 90.00, 90.00
Resolution (Å)	46.36–1.88 (1.95–1.88) *	46.10–2.58 (2.67–2.58)
*R*_merge_ [%]	0.12 (0.88)	0.17 (1.37)
*I* / σ*I*	9.46 (1.81)	14.41 (1.88)
CC_1/2_	0.99 (0.76)	0.99 (0.72)
Completeness (%)	98.70 (97.04)	99.92 (99.96)
Redundancy	5.9	13.2
**Refinement**		
Resolution (Å)	46.36–1.88	46.10–2.58
No. reflections	148,800 (14582)	27,410 (2668)
*R*_work_ / *R*_free_	19.10 / 21.92	20.02 / 23.09
No. atoms	12958	6089
Protein	11662	5945
Ligand/ion	161	85
Water	1135	59
*B*-factors		
Protein	39.96	52.36
Ligand/ion	46.09	50.86
Water	39.27	40.13
R.m.s. deviations		
Bond lengths (Å)	0.011	0.005
Bond angles (°)	1.21	0.79

* Statistics for the highest-resolution shell are shown in parentheses.

## Abbreviations

ADP	Adenosine diphosphate
ATP	Adenosine triphosphate
cMD	Classical molecular dynamics
cryoEM	Cryo-electron microscopy
MM/GBSA	Molecular mechanics/generalized-born surface area
MST	Microscale thermophoresis
P_i_	Inorganic phosphate
rmsd	Root mean square deviation
SMD	Steered molecular dynamics
TMD	Targeted molecular dynamics
